# Health risks and mitigation strategies from occupational exposure to wildland fire: a scoping review

**DOI:** 10.1186/s12995-021-00328-w

**Published:** 2022-01-04

**Authors:** Erica Koopmans, Katie Cornish, Trina M. Fyfe, Katherine Bailey, Chelsea A. Pelletier

**Affiliations:** 1 Health Research Institute, University of Northern British Columbia, 3333 University Way, Prince George, BC V2N 4Z9 Canada; 2 Northern Medical Program, University of Northern British Columbia, 3333 University Way, Prince George, BC V2N 4Z9 Canada; 3 School of Health Sciences, University of Northern British Columbia, 3333 University Way, Prince George, BC V2N 4Z9 Canada

**Keywords:** Wildland fire, Occupational health, Occupational exposure, Mitigation, Prevention, Scoping review, Wildfire, Wildland firefighter

## Abstract

**Objectives:**

Due to accelerating wildland fire activity, there is mounting urgency to understand, prevent, and mitigate the occupational health impacts associated with wildland fire suppression. The objectives of this review of academic and grey literature were to:

1. Identify the impact of occupational exposure to wildland fires on physical, mental, and emotional health; and
2. Examine the characteristics and effectiveness of prevention, mitigation, or management strategies studied to reduce negative health outcomes associated with occupational exposure to wildland fire.

**Methods:**

Following established scoping review methods, academic literature as well as government and industry reports were identified by searching seven academic databases and through a targeted grey literature search. 4679 articles were screened using pre-determined eligibility criteria. Data on study characteristics, health outcomes assessed, prevention or mitigation strategies studied, and main findings were extracted from each included document. The results of this scoping review are presented using descriptive tables and a narrative summary to organize key findings.

**Results:**

The final sample was comprised of 100 articles: 76 research articles and 24 grey literature reports. Grey literature focused on acute injuries and fatalities. Health outcomes reported in academic studies focused on respiratory health (*n* = 14), mental health (*n* = 16), and inflammation and oxidative stress (*n* = 12). The identified studies evaluated short-term outcomes measuring changes across a single shift or wildland fire season. Most research was conducted with wildland firefighters and excluded personnel such as aviation crews, contract crews, and incident management teams. Five articles reported direct study of mitigation strategies, focusing on the potential usage of masks, advanced hygiene protocols to reduce exposure, fluid intake to manage hydration and core temperature, and glutamine supplementation to reduce fatigue.

**Conclusions:**

While broad in scope, the evidence base linking wildland fire exposure to any one health outcome is limited. The lack of long-term evidence on changes in health status or morbidity is a clear evidence gap and there is a need to prioritize research on the mental and physical health impact of occupational exposure to wildland fire.

**Supplementary Information:**

The online version contains supplementary material available at 10.1186/s12995-021-00328-w.

## Background

Across the globe, wildland fire (also known as wildfire or bushfire) activity continues to escalate. Climate change, land use change, and fire exclusion contribute to increased wildland fire activity [1, 2]. Accelerating wildland fire activity necessitates a growing number of personnel engaged in direct wildland fire suppression and related operational roles, exposing them to potential health effects of wildland firefighting. Drier conditions due to higher temperatures and less precipitation have increased the length of wildland fire seasons and contributed to more intense, long-burning fires [3], requiring professionals to work longer periods with less opportunity for reprieve and recovery. In anticipation of continued growth in fire activity, it is essential to understand occupational health impacts of wildland fire exposure on the growing workforce to develop appropriate practices and policies to promote health and safety.

### Wildland firefighting personnel

There are several groups involved in fighting wildland fires. Front-line teams include wildland firefighters (also referred to as wildfire fighters, forest firefighters, or fire rangers) involved in primary fire suppression efforts in specific fireline roles and aerial firefighters involved in aviation operations (e.g., pilots and air crews). In addition to these groups, there may be contract personnel such as crews from the forestry industry involved as equipment operators and fallers, and temporary firefighter forces such as military involved to support mop-up situations and other efforts of low to moderate complexity [4]. Related personnel involved in fire suppression may include incident management teams and support personnel at fire bases engaged in logistical operations and crew management [4]. In some regions of the world or if wildland fire approaches city limits, structural firefighters (city/municipal firefighters) may also support wildland fire suppression [5].

### Existing knowledge

Previous reviews have explored the health impacts from wildland fire exposure in the general population [6–8] and among wildland firefighters [8, 9]. Reviews reporting on wildland firefighters identified limited literature on the health effects of occupational exposure to wildland fire [8, 9] and insufficient evidence to make conclusions with regards to cardiovascular health outcomes, chronic respiratory effects, and cancer risk [8, 9]. While acute physiological responses have been investigated, the clinical significance of findings has not been determined [8, 9] and the reviews conclude that evidence for wildland fire being an acute respiratory hazard for wildland firefighters is weak [8, 9]. Existing reviews highlight the need to study clinically significant health outcomes among the wildland firefighting population [8] and recommend longitudinal study of wildland firefighters during and after their career to understand unknown health impacts of cumulative exposure [8–10]. Prevention, mitigation, and management strategies or policies are seldom mentioned or identified in previous reviews and are predicted to be most effective if targeted to specific fireline roles [9, 10]. The most recent literature search reported was a systematic review conducted in January 2017 and limited to three databases [9]. Given the rapid growth in the field, a continual updated search strategy and synthesis of findings is warranted. Similarly, we are not aware of any previous reviews focused on wildland fire that have included a comprehensive grey literature search. Grey literature includes reports from government and non-government organizations presenting data, research, lessons learned, or program evaluations not published in the academic literature [11, 12]. Including grey literature in scoping reviews can capture additional information and findings relevant for policy makers missing from traditional academic avenues [12].

### Rationale and objectives

The objectives of this review were to: 1) identify the health impacts associated with wildland firefighting across the spectrum of health (including physical, mental, and emotional wellbeing); and 2) examine strategies or policies studied to prevent, mitigate, and/or reduce negative health impacts in this sector. A scoping review approach was chosen as it provides a broad overview of the field of research to identify evidence gaps and inform the current types of evidence available [11–14]. This scoping review was designed to answer the following research questions:

1. What are the occupational health impacts across the spectrum of health (including physical, mental, and emotional wellbeing) associated with wildland firefighting?
2. What prevention, mitigation, and management strategies or policies have been examined to reduce the health impacts of wildland firefighting? What are the characteristics and effectiveness of these approaches?

## Methods

### Protocol and registration

A scoping review method [11–14] was used to identify and synthesize research conducted on the health impacts of occupational exposure to wildland fires and the prevention, mitigation and management strategies used in response. This was done in conjunction with a modified Delphi study (to be published elsewhere) to inform the development of research priorities on wildland firefighter health [15]. A protocol for this project was developed, registered in Open Science Framework (osf.io/ugz4), and is previously published [15]. There are no deviations from the published protocol to report. We do note a minor change in co-authorship from the published protocol due to research team changes. This manuscript has been prepared in alignment with the PRISMA extension for scoping reviews (PRISMA-ScR) (see Additional file 1).

### Identifying literature and selecting relevant publications

#### Eligibility criteria

To be eligible for inclusion, articles were required to focus on wildland firefighters or related personnel who experience occupational exposure to wildland fire. Related personnel were defined as incident management teams, aerial firefighters (pilots and air crew), contract personnel or crews from the forest industry, equipment operators, fallers, military, and personnel at fire bases [4]. Studies that included structural firefighters (municipal firefighters) were included if the focus was on engagement in fighting wildland fires. Eligible studies included the concept of wildland fire (either in field, simulation, or prescribed burn) as an occupational exposure (i.e., individuals were exposed as a direct result of their occupation); and reported outcome(s) on either the health effects of occupational exposure to wildland fire, or strategies, tools, policies, or guidelines studied (i.e., directly measured) to prevent, mitigate or limit health effects. Only articles published in the English language were eligible. Academic research papers and grey literature reports from the health sector, government, wildland fire organizations, and related sectors were eligible for inclusion. The review included research studies with quantitative designs (e.g., experimental, quasi-experimental, and observational studies); qualitative; and mixed methods studies. Case studies and case reports, conference abstracts, editorials and opinion pieces, and literature reviews were excluded. No limits were set on publication date. Rationale for these eligibility criteria are described in the published protocol [15].

#### Information sources and search

Seven academic databases were searched from inception onwards: Ovid MEDLINE, Web of Science, PTSDpubs Proquest, Biological & Agricultural Index Plus EBSCO, CINAHL EBSCO, PsychINFO EBSCO, and GreenFILE EBSCO. The search strategy was designed and conducted by a health research librarian (TF) in December 2019 (see Additional file 2). An additional updated search was completed on October 30, 2020. Eight journals were hand searched for potentially eligible articles: Fire, Safety, International Journal of Wildland Fire, Occupational Medicine, Journal of Occupational Health, International Journal of Occupational Medicine and Environmental Health, Environmental Research, and Environmental Health Perspectives. Targeted searches of government forestry and wildfire/wildland fire agency websites, industry websites, and work safety and union websites were conducted to identify relevant grey literature (see Additional file 3). Reference lists of papers meeting inclusion criteria and relevant review papers [6–9] were also reviewed for additional papers. Articles were screened in two stages. First, titles and abstracts were reviewed for eligibility criteria. Second, the full texts of potentially eligible articles were retrieved and reviewed to confirm eligibility. Overall inter-rater agreement was moderate [16] with a weighted Kappa of 0.73. Disagreements between reviewers on inclusion at either stage were resolved by a third reviewer.

### Extracting the data, synthesizing, and summarizing

Data was extracted from each article on study characteristics, health outcomes assessed, prevention and mitigation strategies evaluated, main findings, and conclusions (see Additional file 4). Data extraction was completed independently by a first reviewer, followed by a ‘quality control’ review of the extraction by a second reviewer. No conflicts were identified in the ‘quality control’ review requiring a third reviewer, only supplemental detail was added to the extraction by the second reviewer. Study quality and risk of bias were not assessed, consistent with published scoping review methods [13]. The extracted data was synthesized and is reported using a narrative description and organized to summarize by category (health outcomes, mitigation strategies) and key characteristics (setting, participant job categories). Findings were classified as ‘cross-shift’ if they reported data before and after a single work shift or work tour and ‘cross-season’ if reporting longitudinal data across a single wildland fire season in the jurisdiction of study. Studies reporting cross-sectional, descriptive, or association data are reported as ‘other key findings.’ Job categories were broken down into wildland firefighter - general (contract, government, and volunteer crews and persons), wildland firefighter - specific roles (specific job role or fireline task identified), structural or industrial firefighters attending a wildland fire, general public, other emergency personnel, and not applicable.

## Results

**Fig. 1:**
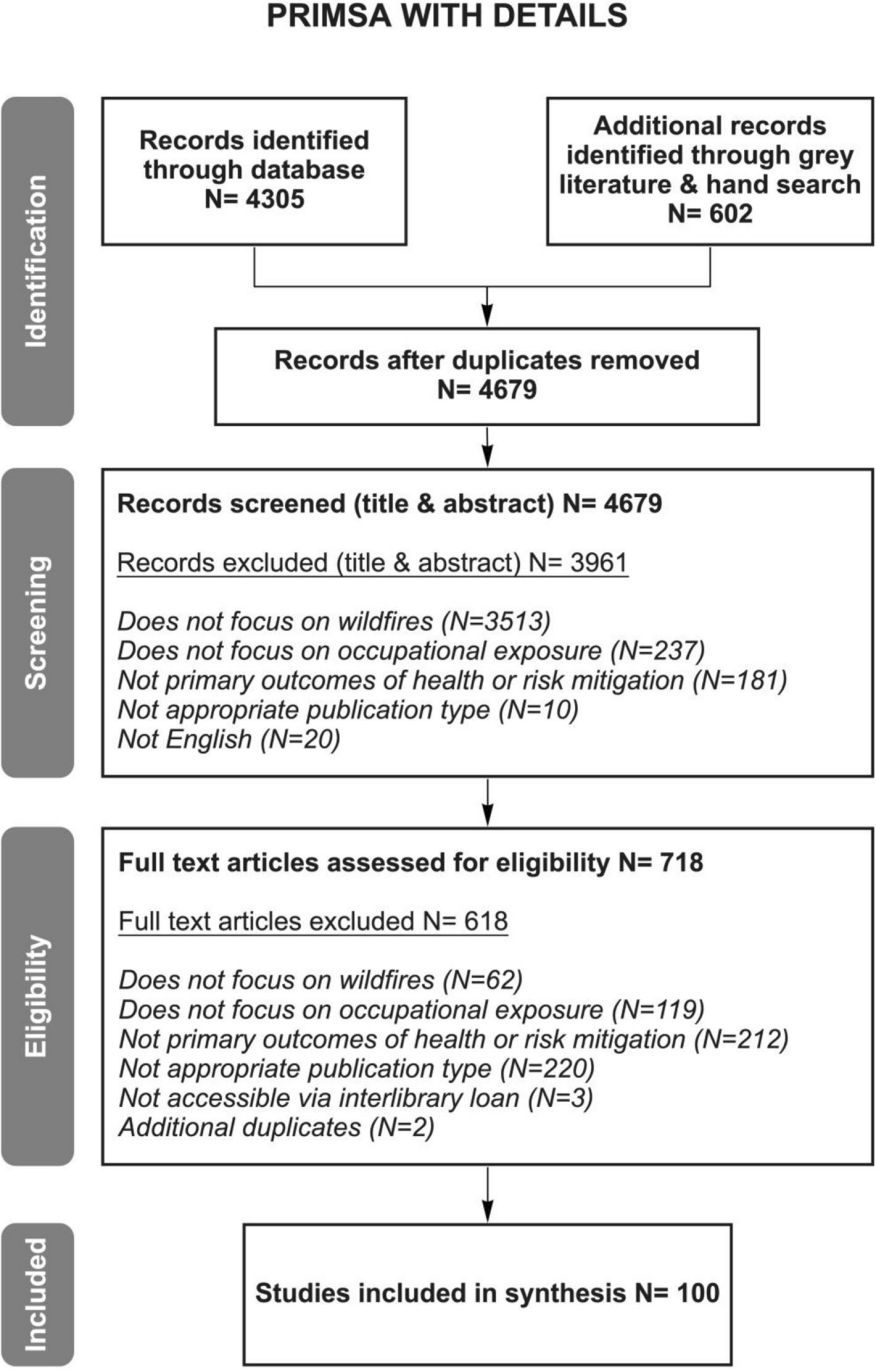
PRISMA Flow Diagram

Our search identified 4679 articles after duplicates were removed, of which 100 articles met the eligibility criteria. The flow diagram of article screening and inclusion is presented in Fig. 1.

### Characteristics of included articles

The final sample was comprised of 76 research articles and 24 grey literature reports (Table 1). Included articles were published between 1985 and 2020. The majority of academic literature was from the USA (*n* = 37) and Australia (*n* = 23). All grey literature publications originated from the USA and Australia. Most research articles used a cross-sectional design (*n* = 39) or prospective cohort (*n* = 20). Health outcomes were measured cross-shift in 20 studies; changes cross-season were measured in nine studies, and three studies collected data both cross-shift and cross-season [17–19]. The majority of included grey literature reports described analyses and summaries of retrospective data (*n* = 23).

**Table 1:** Characteristics of included articles

Characteristic	All included articles (***n*** = 100)	Academic Literature (***n*** = 76)	Grey Literature (***n*** = 24)
**Year of Publication**			
1980s	6	6	0
1990s	5	5	0
2000s	17	9	8
2010s	63	47	16
2020	9	9	0
**Country**			
USA	59	37	22
Australia	25	23	2
Canada	7	7	0
Multi-national	3	3	0
Greece	3	3	0
Israel	1	1	0
Italy	1	1	0
France	1	1	0
**Primary Health Outcome Reported**			
Acute injuries & fatalities	29	8	21
Mental health	16	13	3
Respiratory	14	14	0
Inflammation & oxidative stress	12	12	0
Hydration	6	6	0
Cardiovascular	6	6	0
Fatigue & sleep	7	7	0
Hearing	2	2	0
Temperature regulation	5	5	0
Mercury toxicity	1	1	0
Nutrition	2	2	0
**Study Design**			
Cross-sectional	40	39	1
Data summary or report	23	0	23
Prospective cohort	20	20	0
Experimental	11	11	0
Retrospective	5	5	0
Qualitative	1	1	0
**Participant Job Category**			
Wildland firefighter (general)	84	62	22
Wildland firefighter (specific roles)	2	2	0
Structural or industrial firefighters^a^	7	7	0
General public^b^	4	4	0
Other emergency personnel	2	1	1
Not applicable ^c^	1	0	1
**Study Fire Setting**			
Active wildland fire suppression	46	45	1
Prescribed burn	10	10	0
Simulation	12	12	0
Fire setting not applicable^d^	32	9	23

^a^career or volunteer structural or industrial firefighters attending wildland fires

^b^participants matched to similar age/gender/fitness level of wildland firefighters

^c^simulation study

^d^wildland fire exposures but no described context (e.g., national surveys of wildland firefighters)

Participants in included studies were career and volunteer wildland firefighters (*n* = 84), career or volunteer structural or industrial firefighters attending wildland fires (*n* = 7) or a combination of both (*n* = 19). In six studies participants consisted of control participants (i.e., healthy students, police, and military personnel) in simulated wildland fire activities. Research described in academic articles most commonly took place in active fire suppression (e.g., field) settings (*n* = 46).

Five research articles [20–24] and no grey literature reported on studied mitigation strategies. One grey literature report provided information about critical incident management [25]. Wildland firefighting acute injuries and fatalities were the most reported health outcomes across all articles, this was due to multiple annual fatality grey literature reports (*n* = 18).

### Health outcomes assessed and reported

The included articles (academic and grey literature) were categorized and summarized based on the primary health outcome(s) reported (Table 2).

**Table 2:** Summary of key findings based on health outcome and measurement period

Health Outcome	Key Findings: cross-shift	Key Findings: cross-season	Key Findings: other
Respiratory Health	• Significant decrease in FEV_1_ and FVC [17, 26–28] • No significant change in FEF_75_, PEF, and FEV/FVC [27] • Respiratory symptoms were observed in some studies to increase post-shift [26, 29], but in other studies, no significant change was observed [17] • Post-shift exposure declines in PEF (lesser extent FEV_1_ and FVC) [30]	• Significant decrease in spirometry scores FEV_1_ [18, 31, 32], FEF_25_ [17], FEF_50_ [17], MEF [17], FVC [31, 32], FEF_25–75_ [32] • No significant change in spirometry values [17, 26], respiratory symptoms [17], or sputum levels [26] • Significant increase in respiratory symptom scores [26, 31]	• No significant associations: previous exposure and lung functioning [24, 34], and smoker vs. non-smoker lung function [28] • Significant associations: upper and lower respiratory tract symptoms and FEV_1_ values [26], lung functioning and allergies [27], levoglucosan concentration and FEV_1_ values [36], and firefighting and overall decreased lung function [33]
Cardiovascular Health	• None reported	• Significant increase in LDL, cholesterol, and globulin [38]	• Significant associations: experience in wildland firefighting and hypertension [106], firefighting and risk of lung cancer and cardiovascular disease mortality [19], levoglucosan, particulate matter exposure, and oxidative stress leading to arterial stiffness [37] • No significant associations: between FEV_1_/FVC % and oxidative stress or levoglucosan concentrations [37], wood smoke exposure and vascular vasomotor or fibrinolytic function [39]
Mental Health (PTSD)	• None reported	• None reported	• PTSD symptoms remained long-term after disaster [41, 42], the most common psychological impairment among firefighters [43] • Significant curvilinear relationship between post-traumatic symptoms’ severity and post-traumatic growth [44] • Permanent positions associated with lower risk of PTSD [45] • Increased fear of death and insomnia associated with increased PTSD [45] • Coping mechanisms of minimization and blame were associated with increased PTSD [107]
Mental Health (Other)	• None reported	• None reported	• Intensity of disaster distress is positively associated to the level of psychological impairment [43], the losses sustained in disaster and severity of exposure to disaster were not major determinant of post-traumatic morbidity in firefighters [43, 46] • Association between fatigue, depression, and cytokines [47] • Wildland firefighters have higher levels of suicide risk when compared to non-wildland firefighters [48] • Increased prevalence of ADHD in wildland firefighters compared to general population [53]
Hydration	• Ad libitum drinking and prescribed drinking did not adequately hydrate firefighters throughout shift [22, 23] • Not euhydrated at all times during shift with ad libitum drinking [55] but ad libitum drinking is sufficient for maintaining hydration status pre to post shift [55] and for rehydration [22, 23] • Decreased body weight and total body water pre- to post-shift [56]	• None reported	• Prescribed drinking associated with temporarily lower core temperatures than ad libitum drinking [23] • Wildland firefighters experienced increased core temperature without euhydration [55] • Building fireline increases rate of sweating [59], firefighting is associated with rapid dehydration [59] • Urinary protein excretion associated with intensity of work [57] • Firefighters doubled their fluid consumption in a simulated environment with hot conditions and recorded significantly lower urine specific gravity values (estimating hydration) relative to the lower temperature group (though both groups fell within the ‘hydrated’ range) [110] • Hydration in hot conditions not significantly impaired by restricted sleep [58]
Fatigue and Sleep	• Wildland firefighters reported significantly higher levels of fatigue and decreased alertness with increasing days on deployment and these levels did not improve following a three-day rest period [60] • Wildland firefighters sleep quantity on fire days was significantly less than non-fire days [61] • No differences in sleep efficiency, sleep latency and subjective reports of times woken or sleep quality between non-fire and fire days for wildland firefighters [61] • Self-reported levels of pre- and post-sleep fatigue by wildland firefighters was greater on fire days compared to non-fire days [61] • Decreased sleep quantity and quality associated with high intensity initial attack fire deployment and base work periods [63] • No significant differences between sleep measurements on burn/non-burn days [64], burn day sleep measurements had no association with work shift start time and total sleep time [64]	• None reported	• Decreased gastrointestinal damage, subjective fatigue and perceived exertion associated with glutamine supplementation [22] • Firefighters not under additional thermal strain when working while sleep restricted [58] • Sleep environment, shift duration, and shift start times were associated with reduced sleep time [61] • Sleep measurements decreased and sleep efficiency increased with restricted sleep, slow wave sleep did not change significantly with restricted sleep [62] • No significant association between sleep and hot daytime temperatures [62]
Acute Injuries	• None reported	• None reported	• Injuries more likely to be severe during peak/late season in comparison to early season [65] • Most common causes of injuries: slips/trips/falls [65–67], • Most common injuries sprains/strains [65–67], injuries of lower back/knee/foot/ankle [67] • 20% of firefighters thought their injury was preventable [67] • Majority of injuries occur in rocky, mountainside terrain [67] and are classified as minor [68] • Significant relationship between peak incident management level, person-days of exposure, and resistance of fire to control and odds of at least one injury [69] • Significant associations: an increase in fire complexity and a decrease in incident rate [69], likelihood of injury and increased age, increased neuroticism, decreased openness, and history of injury [70], experience and decreased likelihood of injury [70], reduction in entrapment rates and safety culture shift [71] • Significant predictor of injury is high job stress [70] • Transportation is high-risk [72] • Engine/chainsaw operations had highest incident rates [72]
Fatalities	• None reported	• None reported	• Significant association with death and aviation, vehicles, medical events, and entrapments/burn overs [74] • Leading cause of aircraft crashes resulting in death include aircraft failure, loss of control, failure to clear terrain/water/objects and hazardous weather [75] Yearly Reports: • Four+ deaths of state/federal (USA) wildland management agency personnel on average at fires each year [76, 77] • Deaths by burns [76, 78–83], inhalation [76, 78], cardiac events [76, 81, 85–87], heat stroke [76], vehicle strike [76], strike tree/boulder [76, 78, 80–82, 83, 86–88], equipment failure [79], aircraft crashes [77, 79, 83–85, 87, 89], vehicle crashes [77, 80–86, 88, 89], parachute failure [80], fatal fall [87], chainsaw [81] • 23% of fire ground deaths at wildland fires [70] • Most severe multiple-fatality incidents occur at wildland fires [76, 78, 80] • Causes of death include heart attacks, vehicle accidents, other medical causes, burn overs, aircraft accidents, falling trees/snags/rocks [90, 91]
Inflammation and Oxidative Stress	• Urinary 1-hydroxypyrene correlated with estimated exposure after 48 h [5] • Mean increase in 1-hydroxypyrene in urine samples collected post shift in 76% of participants [20] • Dermal exposure: absorbed polycyclic aromatic hydrocarbons increased in urine samples post shift [20] • Firefighters with drip torches: increase in IL-8, C-Reactive Protein, and serum amyloid [92] • Significant positive association of IL-8 and segmented neutrophils cross-shift [92] • Significant changes in pH, 8-isoprostane and pentraxin-3 [93], marginal increase in 8-isoprostane on burn days [95] • Significant increased airway and systemic inflammation after acute exposure [96] • Significant increase in IL-6 and IL-8 and significant decrease in IL-10 after 12 h of fire suppression [97] • Significant increase in sputum granulocytes post-shift [98] • Significant increase in IL-6, IL-8, and monocyte chemotaxic protein [98] • No significant changes in H_2_O_2_, protein D or myeloperoxidase post wood smoke exposure [93], urinary 1-hydroxypyrene or pulmonary function after pile burns [99], malondialdehyde or 8-oxo-7,8-dihydro-2-deoxyguanosine, except for 8-oxo-7,8-dihydro-2-deoxyguanosine levels for firefighters with under 2 years of experience [100]	• None reported	• Increased scores on the Skin Exposure Mitigation Index significantly related to decrease 1-hydroxypyrene [5] • Additional hygiene measures are effective in reducing dermal polycyclic aromatic hydrocarbons exposure and contamination [20] • Significant association between sputum macrophages with phagocytosed particles and circulating band cells [98] • Firefighting significantly associated with an increased level of basal DNA damage [101] • Increased IL-6 in the morning significantly associated with increased daily cortisol [102]
Hearing	• None reported	• None reported	• Noise exposure regularly exceeds occupational limits [103], • Highest noise exposure from chainsaws, chippers, and masticators [103] • 54% of noise exposure exceeds recommendations [94] • Limited hearing protection use, minimal training on hearing protection [94]
Temperature Regulation	• No significant change in heart rate, skin temperature, rectal temperature, or sweat rate pre- to post-shift [109]	• None reported	• Measures of thermal stress (core temperature, skin temperature and thermal sensation) were significantly higher in participants exposed to higher temperatures while performing wildland firefighting tasks in a simulated environment [110] • Evidence of a multiplying effect for mean skin temperature where wildland firefighters exposed to higher temperature work environment had greater mean skin temperature with each additional work task circuit [110] • Significant increase in skin temperature but no significant increase in cardiovascular or thermal strain while working in-field [111] • Job tasks of higher physical exertion associated with greater changes in core temperature [112]
Mercury Toxicity	• None reported	• None reported	• No significantly elevated levels of mercury toxicity in wildland firefighters [104]
Nutrition	• Sleep restriction and heat did not impact feelings of hunger and fullness across the day, and did not lead to greater cravings for snacks [113] • Discrepancy between total energy expenditure and total energy intake [114]	• None reported	• Wildland firefighters required to work in hot conditions while sleep restricted more likely to consume food between 12:30 and 14:30 h [113]

#### Respiratory health

Fourteen academic articles described respiratory health related outcomes [17, 18, 24, 26–36, 105]. Changes in respiratory health were commonly observed using spirometry measures of lung function or capacity. Respiratory health outcomes included decreases in forced expiratory volume in one second (FEV_1_) and forced vital capacity (FVC) post-shift during prescribed burns [17, 27], in field settings [26, 28], following a lab simulated wildland fire exposure [24], and post-wildland fire [26, 31, 32] and prescribed burn seasons [17]. Other spirometry measures including forced expiratory flow (FEF_75_), and FEV/FVC ratio did not change after a single prescribed burn shift, although there was evidence of cumulative impact after multiple prescribed burn days [27]. Changes in peak expiratory flow (PEF) were mixed with declines post-field exposure in one study [30] but no significant change in another study during a prescribed burn [27]. Respiratory symptoms were found to increase post-season (May – October) [26] and in a shorter 8-week timeframe [31], but this was not consistent when observed across a prescribed burn season [17]. One study found a significant association between upper respiratory tract symptoms and FEV_1_ [26]. Recovery of some short-term changes in lung function were observed by the end of a summer wildland fire season [26] or during annual assessments [17], although another study noted persistent decreases in lung function 3-months post-season [28]. Similarly, two cross-sectional studies demonstrated no significant association between exposure to wildland fire smoke and lung function measures in a previously unexposed population [24, 34].

#### Cardiovascular health

Six academic articles reported on cardiovascular health related outcomes [19, 37–40, 106]. There were no studies that evaluated changes in cardiovascular health cross-shift, and one evaluating changes cross-season, noting an increase in low density lipoprotein (LDL) and total cholesterol [38]. In a cross-sectional self-report survey, one study found a relatively high prevalence of hypertension (13%), elevated cholesterol (13%) and heart arrhythmia (3%) among people with experience working as a wildland firefighter; these cardiovascular risk factors were positively associated with the number of years employed [106]. A second cross-sectional study assessed the association between smoke exposure from wildland fire, oxidative stress, and arterial stiffness [37]. This study found the mean aortic augmentation index (an indirect measure of systemic arterial stiffness collected using pulse wave velocity) was higher for participants with elevated oxidative stress scores [37]. Oxidative stress was associated with increased levels of urinary levoglucosan (a biomarker measure of smoke exposure), suggesting a potential relationship between wildland fire smoke and vascular function [37]. Based on prospective modelling from particulate matter exposure during different field scenarios, Navarro et al. [19] estimated increased risk of cardiovascular disease among short and long wildland fire season lengths, and at various career durations, with risk increasing incrementally based on exposure length.

#### Mental health

Thirteen academic articles and three grey literature reports described mental health related outcomes [25, 41–54, 107]. None of the included studies evaluated changes in mental health outcomes cross-shift or cross-season. One study identified post-traumatic stress disorder (PTSD) as the most common psychological issue among wildland firefighters based on self-report survey 4- (84%) and 11-months (79%) after exposure to a major wildland fire [42]. Another study identified a decrease, but not elimination in psychiatric impairment (e.g., PTSD) up to 7 years following a disaster although there were variations in recovery of psychological distress among participants [41]. One study found subsequent psychiatric impairment (e.g., PTSD) following wildland fire exposure appeared to be related to the intensity of the disaster-associated distress rather than exposure to the disaster itself [43]. Wildland firefighters were reported to have higher levels of suicide risk when compared to non-wildland firefighters which may be attributed to a reduced sense of belonging when deployed away from family and friends [48]. In a cross-sectional survey, one study found a relatively high prevalence of attention-deficit hyperactivity disorder among wildland firefighters (approximately 19.5%) [53].

#### Hydration

A total of six academic articles reported on hydration related health outcomes [22, 23, 55–57, 59]. Studies reporting on various hydration interventions found insufficient hydration pre-shift to post-shift from ad libitum drinking (i.e., drinking when thirsty), prescribed drinking, and pre-shift bolus drinking during in-field [22, 23] and prescribed burn shifts [55]. One study demonstrated decreased body weight and total body water pre-to post-shift in wildland firefighters after a 5-day deployment when compared to recreationally active controls [56]. Compared to the workday average, an increased rate of sweating has been observed during the task of building a fireline, contributing to rapid dehydration during experimental wildland fire as sweat loss was not replaced [59]. In cross-sectional studies, findings demonstrate an increase in core temperature without proper hydration during prescribed burn operations [55]. In a field setting, prescribed fluid consumption resulted in significantly lower core temperatures compared to ad libitum drinking, however no difference was seen in cardiovascular strain [23]. In a simulated wildland fire environment, participants exposed to hot conditions recorded significantly lower urine specific gravity values (used to estimate hydration) relative to the lower temperature group despite increased fluid consumption (though both groups fell within the ‘hydrated’ range) [110]. Ad libitum drinking and self-regulation by firefighters was found to be adequate for facilitating rehydration post-shift [22, 23].

#### Fatigue & Sleep

Seven academic articles reported on fatigue and sleep related health outcomes [21, 58, 60–64]. Measures of decreased sleep quantity and quality were associated with wildland firefighting in field situations [60–63]. None of the included studies evaluated changes cross-season. One study reported progressively higher levels of objective fatigue, suboptimal sleep, poorer performance on cognitive tests, and increased sleepiness during 14 consecutive days of field work on a fireline; these levels did not improve following a three-day rest period [60]. There appears to be differences in sleep quantity and quality between planned burn shifts and work tour deployments to wildland fires. Two studies of wildland firefighters deployed to active fires found that sleep time and quality on fire days was significantly less than non-fire days [60, 61]. Another study during active fire season found suboptimal sleep during non-fire base work conditions and more frequent suboptimal sleep quality and quantity during high intensity initial attack deployments [63]. Sleep environment, shift duration, and shift start times were associated with reduced sleep time in-field [61]. In contrast, there were no significant differences in sleep quality and quantity reported between burn and non-burn days for prescribed burns [64]. Sleep measures taken on planned burn days were found to have no association with shift start time or total sleep time [64].

#### Acute Injuries & Fatalities

Six academic articles and three grey literature reports described outcomes related to acute injuries [65–73]. Four studies reported on a retrospective analysis of United States government safety data over 4 years [65–67, 69] and concluded it was more likely injuries would be severe if they occurred during peak or late season when compared to those early in the season. Mechanism of injury was associated with wildland firefighter age, year, and season [65]. The most common causes of injuries were slips, trips, and falls [65, 66] and injuries sustained by equipment, tools, or machinery [65]. The most frequently reported injuries were sprains or strains [65–67], and lower back, knee, or foot / ankle injuries [67, 68]. Engine crew workers experienced the greatest number of injuries and those working directly on the fireline had increased odds of sprains or strains [66]. Most wildland firefighters sustained at least one injury or illness over a retrospective analysis of five fire seasons [67]. The overall mean injury rate for wildland firefighters was 13.2 per 10,000 person-days [69]. There was an association between increased fire complexity and rates of injury, fires that were resistant to control had increased odds for injury [69]. Injury data analysed from Ontario, Canada identified an inverse relationship between likelihood of injury, increased age, and experience [70].

Two academic and eighteen grey literature papers reported on fatalities [72–91]. There was a significant association between death and aviation related incidents, vehicle incidents, and entrapments (i.e., situations where firefighters become trapped by fire) [74]. The United States Fire Service reported four or more deaths of state/federal wildland personnel at wildland fires each year between 2003 and 2017 [76–89]. The most severe multiple-fatality incidents occurred on the fireline [76, 78, 80]. Causes of death included heart attacks, vehicle accidents, other medical causes, burn overs, aircraft accidents, and falling trees [75, 88]. A shift in safety culture was associated with a reduction in entrapment rates [71].

#### Inflammation & Oxidative Stress

Twelve academic articles reported on inflammation and oxidative stress related outcomes [5, 20, 92, 93, 95–102]. Studies reported an increase in airway and systemic inflammation after acute exposure to wildland fire [96]. This includes increases in IL-6 and IL-8 in field [96, 97] and at prescribed burn shifts [92], and decreases in IL-10 after 12 h (post-shift) of fire suppression [97]. At prescribed burns, there was no association between dose of PM_2.5_ (based on fireline task exposure) and inflammatory biomarkers [92], although an exposure-response relationship was observed between a black carbon measure and inflammation [92]. Other studies demonstrate increased basal DNA damage among wildland firefighters when compared to matched controls; oxidative DNA damage was positively associated with years of firefighting experience [101]. Two studies identified urinary 1-hydroxypryrene (a surrogate measure of polycyclic aromatic hydrocarbons, PAHs) correlated with respiratory exposure to smoke in-field and increased post-shift [5, 20], although another study demonstrated relatively low particulate and PAH exposure with no acute change in urinary 1-hydroxypryrene after prescribed pile burning [99]. Urinary 1-hydroxypryrene was significantly lower the morning after compared to the end of a shift at an active wildland fire [20]. One study demonstrated an increase in granulocytes, macrophages and white blood cells post-shift measured in-field, suggesting a pulmonary and systemic inflammatory response [98].

#### Hearing

Two academic articles reported on hearing-related outcomes [94, 103]. No long-term hearing-related outcomes were reported in the included literature. Both studies used a cross-sectional design to measure noise exposure across wildland firefighting roles and reported that exposure regularly exceeded occupational limits, posing a risk for noise induced hearing loss [94, 103]. The largest exposure occurred from chainsaws, chippers, and masticators [103]. One study asked participants if they received annual audiograms (hearing tests) [94], yet neither study evaluated noise induced hearing loss cross-season.

#### Temperature regulation

Five academic articles reported on temperature regulation [108–112]. During a short experimental fire work shift, no significant changes were found in rectal temperature, skin temperature, or sweat rate regardless of work duration or ambient temperature [109]. A second simulation study reported conflicting results indicating duration of task and ambient temperature influenced skin temperature and thermal sensation, with significant increases measured in participants exposed to higher ambient temperatures [110]. One field study reported increases in skin temperature [111]. In both simulated and field settings, increases in core temperature have been reported [110–112]; job tasks requiring higher levels of physical exertion were associated with greater increases in core temperature [110, 111] but may be moderated by duration of task [110]. Across experimental and field settings, it was found that firefighters were able to manage temperature regulation by self-pacing and modifying activities or duration of task to prevent thermal strain [109–111].

#### Nutrition

Two academic articles described nutrition-related outcomes [113, 114]. No cross-season studies of nutrition related outcomes were identified, and no study examined nutritional deficiencies. The first study reported a discrepancy between total energy expenditure and total energy intake cross-shift indicating feeding practices and rations likely do not provide sufficient fuel for the firefighting assignments [114]. The second study used a simulated wildland firefighting environment to examine the impact of sleep restriction and heat on feelings of hunger and fullness across the day [113]. Sleep restriction and heat did not impact total energy consumption, feelings of hunger, or cravings [113]. Wildland firefighters required to work in hot conditions with restricted sleep were more likely to consume snacks between the hours of 12:30 and 14:30, which could be useful for ensuring sufficient energy intake [113].

### Prevention, mitigation, and management strategies

Five studies reported mitigation strategies. The first study used a simulation lab to evaluate types of protective filters on respiratory symptoms and function: particulate only, particulate/organic vapor, and particulate/organic vapor/formaldehyde. A higher proportion of participants in the particulate mask group and particulate/organic vapor mask group reported respiratory symptoms compared to particulate/organic vapor/formaldehyde filter group [24], although these mask filters were not evaluated in-field for feasibility or user feedback. Cherry and colleagues evaluated respiratory protection (N95 and P100 masks) and improved skin hygiene on PAH exposure in a field setting [20]. Additional hygiene measures of showering with soap and donning clean clothing immediately after returning to base were effective in reducing dermal PAH exposure and contamination [20]. Study findings suggest PAH exposure may have a cumulative effect as post-shift levels did not return to pre-shift levels overnight.

The third study examined the effect of glutamine supplementation (0.15 g/kg body weight pre-and post-exercise) on inflammation and fatigue in a simulated wildland fire scenario (relevant occupational exercises in a heat chamber at 38 °C) compared to a placebo condition over two subsequent days. Results indicate reduced subjective fatigue, reduced perceived exertion, and upregulation of a heat shock response on the second day of simulated work [21]. Findings suggest improved recovery from intensive work in a heated environment and potential reduced risk of injury with glutamine supplementation, although it is not known if these findings extend to a field environment where environmental exposures and work tasks are unpredictable.

Finally, two studies evaluated strategies to address dehydration among wildland firefighters in-field. One study assessed the effectiveness of ad libitum drinking (self-selection of beverage type, timing, volume) in comparison to a pre-shift bolus (500 mL) of water in addition to ad libitum drinking [22], finding no significant differences in core temperature, hydration status, activity, heart rate, or total fluid intake between the ad libitum group and the pre-shift bolus group [22]. Similarly, another study examined the effectiveness of a prescribed drinking intervention (600 mL of water plus 600 mL of sports drink per hour) in comparison to ad libitum (self-selected timing, volume, type of beverage) drinking [23]. Prescribed drinkers consumed twice as much fluid as ad libitum drinkers but did not meet their prescribed fluid intake target reporting it was not needed based on thirst. No differences in plasma sodium or total hydration were found between the two groups after a single shift, however prescribed drinking contributed to reduced core temperature post-shift [23].

## Discussion

Wildland fire research is a fast-growing field of study. Since the publication of previous comprehensive reviews [8, 9] there has been new literature across health outcomes with a focus on inflammation and oxidative stress, fatigue and sleep, and mental health. Wildland firefighting research continues to grow, but there is still nowhere near the amount of evidence on health risks and impacts as there is for structural firefighters and other first responders [105, 115, 116].

### Data on long-term occupational health impacts associated with wildland firefighting is limited

Overwhelmingly the focus of research is on health risks of wildland fire exposure in the short-term, over the course of a single shift or a season, rather than long-term health outcomes over multiple seasons or a career. For example, while changes in respiratory health were observed both cross-shift [17, 26, 27, 29, 30] and cross-season [18, 26, 31, 32], no studies explored the health effects of exposure to wildland fire smoke on chronic disease endpoints such as asthma or chronic obstructive pulmonary disease (COPD). Although some studies identified changes in risk factors for chronic disease, such as increased arterial stiffness [37], hypertension [106], and decreased respiratory function [33], we are unable to conclude if occupational exposure to wildland fire is associated with increased morbidity, which is consistent with a previous systematic review [9]. Based on the estimated exposure-response relationship, modelling suggests wildland firefighters are at increased risk of lung cancer and cardiovascular mortality [19], although this finding has yet to be confirmed in a prospective cohort.

The focus on short-term health impacts is likely the result of challenges in conducting long-term studies with this population and the unpredictability of fire activity. In the wildland firefighting workforce, many of the employees are auxiliary, meaning they are hired for the summer fire season (spanning April – September in British Columbia and much of North America) and may or may not return in subsequent seasons. This creates challenges for tracking participants over multiples seasons. To address the challenges of seasonal employment and an auxiliary workforce, it may be necessary to conduct a retrospective analysis or chart review to determine if people who worked for multiple seasons have increased risk of illnesses like asthma, COPD, cancer, and cardiovascular disease. As wildland fire seasonality changes, it becomes particularly important to understand if short-term changes resolve after the season with periods of no exposure or if they have a cumulative impact, as extended fire seasons reduce downtime and opportunity to recover. Similarly, due to differences across jurisdictions in exposure, fire suppression strategies, and workforce, without a fulsome body of evidence or multi-jurisdictional studies, it is challenging to make global statements regarding health risk or effective mitigation strategies as exposures vary by fireline task, suppression strategies, and fuel type [10].

Mental health has been studied in published evidence, but research has primarily focused on PTSD [41–43] with limited attention to other aspects of mental and emotional health. Like research on respiratory health outcomes, there has been limited exploration of long-term cumulative risk over multiple seasons, how people recover across seasons and if there are differing outcomes for staff who work seasonally versus in a permanent (year-round) position. PTSD and mental health risk must be contextualized for wildland firefighting where the trauma exposure differs from the traditional model as applied to other first responders [48].

Sleep and fatigue are less frequently studied in the literature. The cognitive impacts of fatigue have only recently been studied in wildland firefighters and indicate significantly poorer cognitive performance at the end of a 14-day work deployment [60]. The impact of chronic fatigue over the fire season is not reported, although in other populations chronic fatigue has important implications for decision making, mood, and other health outcomes [117, 118]. Current evidence consistently describes decreases in sleep quantity and quality during field deployments. Understanding the impacts of sleep duration and quality on wildland firefighter cognitive function, decision making, and wellbeing is important to determine appropriate mitigation strategies at the individual (e.g., wildland firefighter sleep hygiene) and organizational (e.g., shift or tour length) level.

### Unknown health risks for personnel not engaged in direct fire suppression

Available evidence focuses on front-line wildland firefighters with limited attention to other related personnel (e.g., incident management teams, air support). In a 2019 systematic review, Groot et al. highlighted a lack of research focus on other personnel attributed to the primary focus on cardiovascular and respiratory health impacts [9]. While smoke inhalation or exposure may be less of a risk for those not engaged in direct fire suppression, there are other potentially relevant risks related to mental health, stress, and fatigue yet to be quantified.

### Gap in intervention and mitigation research to reduce negative health outcomes

The currently published academic and grey literature focuses on reporting short-term studies to measure function, injuries, fatalities, or changes in health status across shifts or seasons but minimal effort to manage and reduce this risk. Of the five mitigation studies included in this review, one conducted field testing on the fireline in British Columbia and Alberta, Canada [20], two conducted field testing with Australian and American fire crews in Australia [22, 23], and two were simulated wildland fire environments [21, 24]. The findings of these studies are limited due to the simulated wildland fire settings [24] and the small sample sizes [20, 22, 23]. While these five studies demonstrated early promise for the use of masks and enhanced hygiene protocols post-shift to reduce respiratory and dermal exposure to contaminants, and glutamine supplementation to reduce fatigue, the development and evaluation of mitigation strategies are a critical area for further attention. Currently, use of personal protective equipment is limited among wildland firefighters. Hard hats are typically worn, but wearing masks or respirators is not a regular practice among wildland firefighters [119] and limited hearing protection is worn [94]. It is prudent that future work considers user experience, acceptability, and feasibility of such strategies, as any personal protective equipment including masks, or associated health and safety policy, must be developed based on realistic usage in-field. This is crucial to ensure buy-in, eventual uptake, and to combat organizational resistance to change. In addition to personal protective equipment, other mitigation strategies may include operational modifications to reduce overall smoke exposure, such as moving fire camps away from dust and smoke. Finally, we note that while only five included articles directly evaluated a mitigation strategy, many others discussed possible approaches which are described in the full data extraction table (Additional file 4).

### Strengths and limitations

This review is strengthened by using rigorous, transparent, and systematic methods for the selection, analysis, and synthesis of academic and grey literature. This creates a reproducible approach for any future updates of this review. The major limitation of this review is the inclusion of only English publications. In limiting publications to the English language, we may have missed pertinent research from other regions which also encounter wildland fires, for example Spanish or Portuguese publications from countries such as Mexico, Portugal, and regions of South America. Further, scoping review methodology is focused on the breadth of research in a field. As a result, critical appraisal of the quality of evidence or risk of bias in each primary research study was not assessed. This means we cannot confidently speak to the quality of research and validity of the findings, which will be an important consideration as the field advances. Similarly, our search strategy was designed to capture the breadth of research on health impacts associated with occupational exposure to wildland fire. As such, we may not have captured papers reporting on some physiological outcomes (e.g., heat exposure/temperature regulation, hydration status) tangentially related to health and a more targeted search strategy may be needed to fully synthesize these specific topics.

## Conclusions

While research on wildland firefighting continues to grow, the evidence base on any one particular health issue is limited and synthesis of findings on health risk is inconclusive. The lack of long-term evidence on changes in health status or morbidity is a clear evidence gap and limits the ability to know where prevention and mitigation efforts should be targeted. There is a need to prioritize research on the long-term mental and physical health risks of wildland fire for both wildland firefighters and others engaged in related roles.

## Supplementary Information

**Additional file 1.** PRISMA-ScR Checklist

**Additional file 2.** Search strategy for Ovid Medline

**Additional file 3.** Grey literature search

**Additional file 4.** Complete data extraction of included articles

## Data Availability

Not applicable.

## Abbreviations

FEF: Forced expiratory flow
FEV_1_: Forced expiratory volume in one second
FVC: Forced vital capacity
LDL: Low density lipoprotein
PAH: Polycyclic aromatic hydrocarbons
PEF: Peak expiratory flow
PRISMA-ScR: Preferred Reporting Items for Systematic Reviews and Meta-Analysis Protocols Extension for Scoping Reviews
PTSD: Post-traumatic stress disorder
