# Supplementary material for: Health risks and mitigation strategies from occupational exposure to wildland fire: a scoping review
Source: J Occup Med Toxicol. 2022 Jan 4;17:2. doi: 10.1186/s12995-021-00328-w (PMC8725416; doi:10.1186/s12995-021-00328-w)
Supplement: Supplementary file 2 — Additional file 2. Search strategy for Ovid Medline [file 12995_2021_328_MOESM2_ESM.docx]

Additional File: Medline OVID Search Strategy

**Ovid MEDLINE(R) and Epub Ahead of Print, In-Process & Other Non-Indexed Citations and Daily** 1946 to December 04, 2019

| **#** | **Searches** | **Results** |
| --- | --- | --- |
| 1 | (fire* adj3 (bush* or wild or biomass or forest* or grass* or habitat* or vegetation or ecosystem* or savanna* or agricultur* or wildland)).ti,ab. | 2279 |
| 2 | (bushfire* or forestfire* or grassfire* or wildfire* or smoke pollution event*).ti,ab. | 1972 |
| 3 | ((bush or forest or grass or wild or peat or agrigultur*) adj3 fire*).ti,ab. | 1273 |
| 4 | ((wood* or vegetable or component* or peat) adj3 (smoke or particulate or fire*)).ti,ab. | 2594 |
| 5 | (prescribed adj3 (burn* or fire*)).ti,ab. | 444 |
| 6 | ((fire* or wildfire*) adj3 smoke).ti,ab. | 678 |
| 7 | Wildfires/ | 268 |
| 8 | Smoke/ | 8183 |
| 9 | (fire adj3 suppression).ti,ab. | 317 |
| 10 | 1 or 2 or 3 or 4 or 5 or 6 or 7 or 8 or 9 | 14415 |
| 11 | (firefighter* or firefighting).ti,ab. | 2287 |
| 12 | Firefighters/ | 957 |
| 13 | (fire adj3 (crew* or pilot* or ranger* or fighter* or brigade* or personnel)).ti,ab. | 651 |
| 14 | ((air or contract or attack or initial or hand or unit or engine or helitack or project or rappel) adj3 crew*).ti,ab. | 284 |
| 15 | (parattack or helitack or rapattack).ti,ab. | 0 |
| 16 | (initial adj2 attack).ti,ab. | 741 |
| 17 | (smokejumper* or handcrew* or hotshot*).ti,ab. | 38 |
| 18 | volunteer*.ti,ab. | 187759 |
| 19 | exp Occupational Exposure/ | 61791 |
| 20 | 11 or 12 or 13 or 14 or 15 or 16 or 17 or 18 or 19 | 252158 |
| 21 | (intervention or prevention or policy or policies or guideline* or administrative).ti,ab. | 1535730 |
| 22 | exp Risk Management/ | 289478 |
| 23 | (risk adj3 (management or prevention or intervention)).ti,ab. | 33256 |
| 24 | 21 or 22 or 23 | 1784325 |
| 25 | 10 and 20 | 768 |
| 26 | 24 and 25 | 127 |
| 27 | from 25 keep 1-289 | 289 |
