# Supplementary material for: Health risks and mitigation strategies from occupational exposure to wildland fire: a scoping review
Source: J Occup Med Toxicol. 2022 Jan 4;17:2. doi: 10.1186/s12995-021-00328-w (PMC8725416; doi:10.1186/s12995-021-00328-w)
Supplement: Supplementary file 3 — Additional file 3. Grey literature search [file 12995_2021_328_MOESM3_ESM.docx]

## Additional File: Grey Literature Search Strategy

1. Identified and created a list of government forestry and wildfire/wildland fire agency websites, industry websites, and work safety and union websites.
2. Searched identified websites using key terms for health, prevention, mitigation, and management for any resources, reports, or documents.
3. Also reviewed specific pages on sites that housed resources, reports and documents.
4. Contacted experts in the field (BC Wildfire Service) to identify studies or reports that they were aware of, websites that should be included, and additional sources of grey literature.

| **Agency Website Searched** | **Date Searched (dd-mm-yyyy)** | **# of Documents**  **Identified** | **Date Searched (dd-mm-yyyy)** | **# of Documents**  **Identified** |
| --- | --- | --- | --- | --- |
| Canadian Interagency Forest Fire Centre (CIFFC) | 04-12-2019 | 2 | 12-11-2020 | 0 |
| BC Wildfire Service | 04-12-2019 | 0 | 12-11-2020 | 0 |
| US Fire Service | 09-12-2019 | 0 | 12-11-2020 | 0 |
| Alberta Wildfire | 26-01-2020 | 1 | 12-11-2020 | 2 |
| Government of Saskatchewan Wildfire Operations | 26-01-2020 | 0 | 12-11-2020 | 0 |
| Manitoba Sustainable Development Wildfire Program | 26-01-2020 | 0 | 12-11-2020 | 0 |
| Ontario Ministry of Natural Resources and Forestry | 28-01-2020 | 1 | 12-11-2020 | 0 |
| Nova Scotia Department of Natural Resources Wildfire Management | 28-01-2020 | 0 | 12-11-2020 | 0 |
| Yukon Wildland Fire Management | 28-01-2020 | 0 | 12-11-2020 | 0 |
| Northwest Territories Wildland Fire Operations | 28-01-2020 | 0 | 12-11-2020 | 0 |
| Quebec Societe de Protection des Forets Contre Le Feu | 28-01-2020 | 0 | 12-11-2020 | 0 |
| Australian Authorities Fire Council | 07-03-2020 | Requires Sign-In |  | N/A |
| Bushfire and National Hazards CRC | 07-03-2020 | 9 | 12-11-2020 | 1 |
| Government of New South Wales – NWS Rural Fire Service | 10-03-2020 | 38 | 12-11-2020 | 1 |
| Queensland Fire and Emergency Services – Rural Fire Service | 10-03-2020 | 43 | 12-11-2020 | 0 |
| South Australian Fire and Emergency Services Commission – Country Fire Service | 10-03-2020 | 0 | 12-11-2020 | 3 |
| Tasmania Fire Service | 11-03-2020 | 7 | 12-11-2020 | 1 |
| Department of Environment, Land, Water, and Planning | 13-03-2020 | 8 | 12-11-2020 | 0 |
| Department of Fire and Emergency Services of Western Australia | 14-03-2020 | 21 | 12-11-2020 | 0 |
| Fire and Emergency New Zealand | 14-03-2020 | Requires Sign-In |  | N/A |
| US Department of the Interior’s Bureau of Land Management | 17-03-2020 | 3 | 19-11-2020 | 0 |
| National Parks Service | 18-03-2020 | 1 | 19-11-2020 | 0 |
| Florida Division of Forestry | 18-03-2020 | 1 | 19-11-2020 | 0 |
| California Department of Forestry and Fire Protection (CDF) | 18-03-2020 | 0 | 19-11-2020 | 0 |
| National Wildlife Coordinating Group | 20-03-2020 | 3 | 19-11-2020 | 0 |
| National Interagency Fire Centre | 20-03-2020, 27-03-20 | 30 | 19-11-2020 | 0 |
| Parks Canada | 20-03-2020 | 0 | 19-11-2020 | 0 |
| Emergency Management BC | 20-03-2020 | 0 | 19-11-2020 | 0 |
| FireScience.gov | 20-03-2020 | 3 | 19-11-2020 | 0 |
| Wildfire Today | 20-03-2020 | 28 | 19-11-2020 | 10 |
| Wildland Fire Lessons Learned Centre | 23-03-2020 | 62 | 19-11-2020 | 30 |
| Wildland Fire Lessons Learned Centre Wordpress | 25-03-2020 | 44 | 19-11-2020 | 8 |
| Institut de recherche Robert-Sauvé en santé et en sécurité du travail (IRSST) | 26-03-2020 | 4 | 19-11-2020 | 0 |
| Northern Research Station (NRS) Fire Research | 26-03-2020 | 13 | 19-11-2020 | 0 |
| United States Department of Agriculture Forest Service | 26-03-2020 | 0 | 19-11-2020 | 1 |
| United States Department of Agriculture Treesearch | 27-03-2020 | 0 | 19-11-2020 | 1 |
| The National Institute for Occupational Safety & Health (NIOSH) | 27-03-2020 | 25 | 19-11-2020 | 2 |
| [Wildland Firefighter](https://wildlandfirefighter.com/) | 27-03-2020 | 7 | 19-11-2020 | 0 |
| [Firefighter Nation](https://www.firefighternation.com/) | 27-03-2020 | 1 | 19-11-2020 | 0 |
